# Consideration of Oral Health Within Guidelines and Policy Documents Focused on Nutritional Status and Dietary Intake for Older Adults in Care Homes in the UK: A Scoping Review

**DOI:** 10.1111/ger.70032

**Published:** 2025-12-28

**Authors:** Sachi Makino, Jayne Woodside, Aziza Sallam, Noleen McCorry, Michelle Harvey, Anja Heilmann, Caroline Lappin, Clare McEvoy, Gary Mitchell, Sinead Watson, Ciaran O'Neill, Georgios Tsakos, Paul Brocklehurst, Gerry McKenna

**Affiliations:** ^1^ Queen's University Belfast Belfast Northern Ireland UK; ^2^ University College London London UK; ^3^ Department of Health Belfast Northern Ireland UK; ^4^ Public Health Wales Wales UK

**Keywords:** care homes, diet, guidelines, nutrition, older adults, oral health, policy documents

## Abstract

**Introduction:**

Older care home residents are particularly vulnerable to both malnutrition and chronic dental diseases, such as caries and periodontal disease. While high‐sugar oral nutritional supplements and fortified foods are commonly used to prevent undernutrition, excessive sugar intake increases the risk of dental caries. Despite the well‐established interplay between nutrition and oral health, existing guidelines and policy documents tend to address these aspects separately, resulting in fragmented care. This study aims to examine how oral health is considered within guidelines and policy documents focused on nutritional status and dietary intake for care home residents in the United Kingdom.

**Methods:**

A scoping review was conducted using Arksey and O'Malley's framework. A systematic search identified UK and Irish based guidelines and policy documents addressing both nutrition and oral health in care homes. Documents exclusively covering either nutrition or oral health were excluded. Data were extracted, reviewed and analysed thematically.

**Results:**

Twelve documents were included: five nutritional documents incorporating oral health and seven oral health documents referencing dietary intake. Nutritional documents acknowledged sugar‐related oral health risks but lacked practical caries prevention strategies. Oral health documents emphasised sugar restriction and hygiene but provided limited guidance on balancing nutritional adequacy with oral health preservation.

**Conclusion:**

Current guidelines and policy documents insufficiently integrate oral health and nutrition, resulting in fragmented care. Future policies should adopt an interdisciplinary approach, incorporating evidence‐based dietary and oral health strategies to improve care home residents' well‐being.

## 1 Introduction

With the steadily growing older population and advancements in healthcare, people are living longer lives [1, 2]. This demographic shift has resulted in an increased demand for long‐term care services [3, 4]. As of 2022, the number of people living in care homes across the United Kingdom (UK) was estimated to be approximately 410,400 [5]. According to the most recent government statistics from January 2025, there are approximately 181,965 people receiving support in care homes in England alone, with 130,475 residing in residential care homes and 51,490 in nursing homes [6]. In addition, with changing oral epidemiology, the number of partially dentate older adults has increased [7, 8, 9]. In fact, many care home residents now retain their natural teeth [10]. Retaining healthy natural teeth in later life is structurally, functionally and psycho‐socially beneficial. However, partially dentate older adults are vulnerable to chronic dental diseases, particularly dental caries and periodontal disease, as evidenced by epidemiological studies from a number of countries [8, 11, 12].

A number of previous studies have illustrated that care home residents are more susceptible to dental diseases than older adults living in the community [8, 13, 14]. A number of factors contribute to this situation particularly as older adults in care homes are often dependent on others for oral care, experience difficulties with mobility and manual dexterity, plus the challenges of cognitive decline and dementia [15, 16]. However, the relationship between chronic oral diseases, particularly caries, and high dietary intakes of complex carbohydrates and sugars are also well established [17, 18, 19]. Preventing and addressing malnutrition amongst care home residents poses major clinical challenges, especially given the high prevalence of frailty [20, 21]. As a result, it is common in care homes to provide food and drinks, and oral nutritional supplements (ONSs), containing sugars to boost caloric intake [22, 23, 24]. However, excessive and/or frequent sugar consumption can be a significant risk factor for the development of chronic dental diseases in this environment [25, 26, 27].

Chronic dental diseases have profound negative impacts on older adults' quality of life (QoL), systematic medical conditions and health care costs for individuals and society [13]. Both caries and periodontal disease can give rise to pain, discomfort and negative impacts on oral function (masticatory function, speaking, social interaction) through ultimately causing loss of natural teeth [28, 29, 30]. Reduced masticatory ability may in turn impact dietary eating habits, such as avoiding tough foods that are high in fibre including fruit, vegetables and nuts [31, 32, 33]. Reductions in key nutrients for optimal health and healthy ageing may subsequently occur increasing overall risk for age‐related systemic diseases such as cardiovascular diseases, cancer, chronic respiratory diseases and dementia [34, 35].

Despite the importance of maintaining good oral health for care home residents, evidence clearly illustrates that oral healthcare provision and service in care homes is often poor [10]. This suggests that oral health is a low priority in care home settings, particularly given the potentially significant negative impacts which prescribed diets and nutritional plans can have on remaining natural teeth [10, 22, 24, 26, 36]. Accordingly, the aims of this scoping review was: to identify available guidance where both dietary intake and nutritional status, and oral health are optimised; to identify the gap in guidelines and policy documents focusing on both dietary intake and nutritional status, and oral health for older adults in care homes throughout the UK.

## 2 Material and Methods

To investigate what guidance is available to improve the nutritional status of older adults in care homes and how this guidance considers oral health, a scoping review was completed. The review was conducted according to the Arksey and O'Malley's framework and included published guidelines and policy documents [37].

**FIGURE 1:**
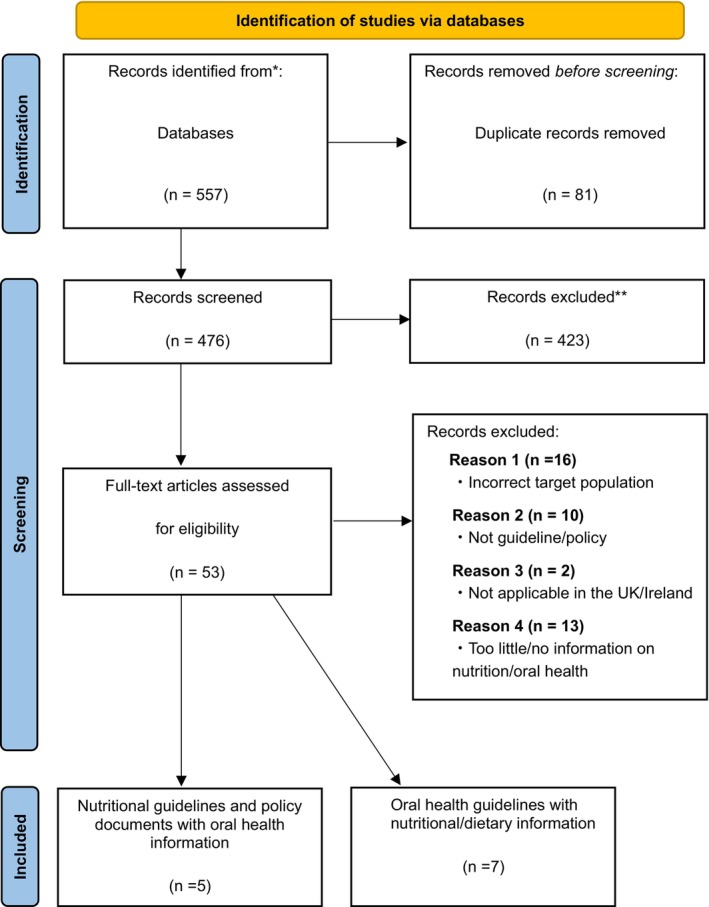
Flow diagram for literature search (PRISMA) [38]. [Colour figure can be viewed at wileyonlinelibrary.com]

The inclusion criteria were that the guidelines and policy documents focused on both nutrition and oral health for residents in care homes in the UK. The exclusion criteria were; (1) Guidelines and policy documents that did not specifically target care home residents—this included documents focused exclusively on community‐dwelling older adults, documents that addressed both community‐dwelling and care home residents without distinction, or documents that did not clearly specify the target population (corresponding to ‘Incorrect target population’ in the flow diagram in Figure 1), (2) the guidelines and policy documents focused solely on nutrition or oral health, and [3] The guidelines and policy documents were not applicable to the UK.

Comprehensive searches were conducted on multiple databases, such as PubMed, MEDLINE, Web of Science, Google and Google Scholar, using the following terms (Table 1): (‘nutritional status’ OR ‘food’ OR ‘drink’ OR ‘meal’ OR ‘eating’ OR ‘oral nutritional supplement’) AND (‘oral health’ OR ‘caries’ OR ‘tooth decay’ OR ‘tooth loss’ OR ‘gum disease’ OR ‘periodontal disease’ OR ‘oral rehabilitation’ OR ‘oral hygiene’) AND (‘older adults’ OR ‘older individuals’ OR ‘old people’ OR ‘elderly’ OR ‘geriatrics’) AND (‘care home’ OR ‘nursing home’ OR ‘group home’) AND (‘UK’) AND (‘guideline’ OR ‘policy’ OR ‘guide’ OR ‘recommendation’ OR ‘strategy’) (Table 1).

**TABLE 1:** The search terms used in the scoping review.

Concept 1	Concept 2	Concept 3	Concept 4	Concept 5	Concept 6
Diet	Oral health	Older adults	Care home	Place	Policy documents
Nutritional status	Oral health	Older adults	Care home	UK	Guideline
Food	Caries	Older individuals	Nursing home	England	Policy
Drink	Tooth decay	Old People	Group home	Scotland	Guide
Meal	Tooth loss	Elderly		Wales	Recommendation
Eating	Gum disease	Geriatrics		Northern Ireland	Strategy
Oral nutritional supplement	Oral rehabilitation				
	Oral hygiene				

The search was conducted in February 2025, identifying guidelines and policy documents published from 1946 to 2024. This start date reflects the default coverage of the Ovid MEDLINE database, which begins in 1946 with the launch of Index Medicus, the precursor to PubMed/MEDLINE. No publication year limits were applied. The initial search was conducted according to the flow diagram illustrated in Figure 1.

Firstly, duplicates were eliminated automatically using the Endnote tool for duplicate detection and manually based on abstract screenings. During this step, one reviewer (SM) charted all the data from the included guidelines and policy documents based on (a) Bibliographic details: Year, issuing authority (s), title, document type and location; (b) Key content (Tables 2 and 3). For ease of cross‐referencing between the text and tables, each included document was assigned a unique identifier: nutritional documents as ‘NTx’ and oral health documents as ‘OHx,’ where ‘x’ denotes the document number in Tables 2 and 3. After this step, the second reviewer (AS) extensively analysed the extracted data to validate its accuracy. Consensus was reached on any discrepancies and necessary corrections by consultation with a third reviewer (GMK).

**TABLE 2:** Summary of nutritional/dietary guidelines and policy documents with oral health information.

ID	Title	Year	Document type	Organization	Location	Content			
						High‐sugar diet in care homes	Caries prevention in care homes	Cross‐referencing of nutritional and oral health guidelines and related resources	The link between nutrition and oral health
NT 1	Best Practice Guidance: FOOD & NUTRITION in Care Homes for Older People Section 1: Why eating and drinking well matters	2019	Guidance	The Welsh Government	Wales	Residents requiring modified diets and in particular fortified diets that require additional foods and/or drinks that may have higher sugar content. Residents with dementia and/or taste change may have a preference for sweeter foods and it will be important to offer food and drinks containing more sugar if they are not eating well.	If consuming foods and drinks high in sugar, have these less often and in small amounts. Dried fruit and fruit juices can be damaging to teeth, so include these as part of meals. Residents who need a modified diet through the use of ONSs, sugary foods or medication will need extra oral care. Poor oral care can result in gum disease, tooth decay and tooth loss.	Gwên am Byth programme, an all Wales programme to help you to provide consistent high quality oral hygiene and mouth care for care home residents, was introduced. Nutrition care plans should be supported by an oral health risk assessment, such as all Wales oral health risk assessment in Gwên am Byth programme.	Oral health and dietary intake are also closely linked – having poor oral health, tooth decay and gum disease can reduce food intake and enjoyment of food, and have a big impact on QoL. Ill fitting dentures are also a key cause of discomfort and they may also indicate previous weight loss, which should be identified when first assessing a resident's needs.
NT 2	Nutritional guidelines and menu checklist for residential and nursing homes	2014	Guideline and Checklist	Public Health Agency (HSC, Belfast)	Northern Ireland	Factors such as increased sugar intake, the use of syrupy medications and ONSs can be associated with people living in care homes, and can compound oral health problems such as caries, periodontal disease and tooth loss.	Foods and drinks that are high in sugar can lead to tooth decay, especially if they are taken too often between meals, therefore they should be limited to mealtimes where possible. Unsweetened fruit juices contain high natural sugars and can therefore contribute to caries. It is better to take it at mealtimes. Additional snacks should be offered to those residents who are nutritionally at risk and require additional calories. Extra attention to oral health is required due to their high sugar content.	No relevant content found	Good oral health is essential for enjoying food. Nowadays, an increasing proportion of older people are retaining their natural teeth. When teeth are maintained in a reasonably healthy state, it can make a significant, positive contribution to an older person's general health in terms of oral function, nutrition and QoL.
NT 3	Care Home Digest—Menu planning and food service guidelines for older adults living in care homes	2024	Guideline	The Association of UK Dietitians (BDA)	England	No relevant content found	This guideline recommended that dentists should be involved when nutrition and hydration care plans were established. When nutrition and hydration care plans are issued, dentists can provide information about oral health, tooth‐friendly food and fluid choices or timing of meals and snacks.	Several oral health guidelines and resources are introduced: ER‐Mouthcare‐for‐Older‐People‐Information‐for‐Carers‐BSG1205upload.pdf (nice.org.uk); Mouth care and oral health for people with dementia UK, YouTube; How to Clean a Denture Animation—Mouth Care Matters, YouTube; Supporting patients hospital who are resistant to mouth care YouTube and Gwên am byth Public Health Wales (nhs.wals)	No relevant content found
NT 4	Eating and drinking well in care: good practice guidance for older people	2018	Guidance	Care inspectorate	Scotland	This guidance highlights the risks of high sugar intake in care homes, particularly its contribution to root decay in older adults. Sugary foods, syrupy medications, and ONSs can increase the risk of tooth decay. Additionally, dry mouth due to polypharmacy or Candida infections may lead individuals to consume sugary drinks or sweets, further exacerbating the issue.	This guidance emphasises oral hygiene and caries prevention in care homes. It recommends regular cleaning of dentures and soft tissues, similar to natural teeth, to prevent plaque buildup. An oral health risk assessment should be conducted upon admission and at regular intervals. Additionally, it references the Caring for Smiles Guide, which advises enhancing mouth care for older adults consuming more sugar due to potential caries risk. The guidance also recommends core training for care home staff, though not solely for caries prevention but for broader oral health management.	This guidance references a Scottish national oral health guideline: the Caring for Smiles Guide for Care Homes, which aims to improve oral health practices in care home settings. It provides guidance on oral hygiene, risk assessments, and oral care strategies for residents. Additionally, it highlights the importance of integrating oral health into broader care home policies and staff training programs.	This guidance emphasises the strong link between oral health, nutrition, and overall well‐being. Good oral health supports nutrition, self‐esteem, and social engagement, while poor hygiene can cause pain, tooth loss, and reduced QoL. Proper chewing and swallowing are essential for a balanced diet and hydration. Additionally, older adults with dementia may struggle with eating and drinking due to swallowing difficulties and dental issues, increasing the risk of malnutrition.
NT 5	Nutritional Guidance for Care Homes 2024	2024	Guidance	Norfolk and Waveney Integrated Care System	England	No relevant content found	This guidance highlights the importance of oral hygiene in care homes, particularly for residents with dentures or partial dentures. It recommends regular mouth washing and teeth brushing to maintain oral cleanliness and prevent plaque buildup. Additionally, care staff should provide daily support for residents' oral hygiene according to their personal care plans. However, the guidance does not explicitly state that these measures are specifically for caries prevention.	This guidance references NICE NG48: Oral Health for Adults in Care Homes as a source for additional guidance on oral health in care homes. However, it does not provide further details on the topic within this document.	This guidance highlights the relationship between oral health and nutrition. It states that poor oral health can negatively impact dietary intake, as difficulties in chewing, swallowing or tasting food may lead to reduced food consumption. Additionally, ill‐fitting dentures or sore gums can restrict the ability to eat certain foods, limiting dietary variety. The guidance also states that poor diets are linked to a higher risk of dental conditions and that inadequate hydration can contribute to poor oral health in older adults.

**TABLE 3:** Summary of oral health guidelines and policy documents with nutritional/dietary information.

ID	Title	Year	Document type	Organization	Location	Content			
						High‐sugar diet in care homes	Caries prevention in care homes	Cross‐referencing of nutritional and oral health guidelines and related resources	The link between nutrition and oral health
OH 1	Guidelines for Oral Health Care for Long‐stay Patients and Residents	2000	Guideline	BRITISH SOCIETY FOR DISABLITY AND ORAL HEALTH	UK	No relevant content found	ONSs prescribed to support nutritional status can present risks to oral health in individuals with natural teeth. Care staff should recognise the factors that pose risks to oral health and apply effective methods to prevent dental caries.	The Nutrition Task Force emphasised the importance of offering consumers clear, practical, and achievable advice on choosing a balanced diet.	The impact of diet and nutrition on both oral and overall health are critical matters that need to be considered. The guideline emphasises the critical role of good nutrition in supporting the health, well‐being and independence of older adults. Conversely, poor oral health in institutionalised populations may lead to issues like difficulty eating, weight loss, dehydration and a decline in physical condition.
OH 2	GUIDELINES FOR THE ORAL HEALTHCARE OF OLDER PEOPLE LIVING IN NURSING AND RESIDENTIAL HOME IN NORTHERN IRELAND	2012	Guideline	GUIDELINES AND AUDIT IMPLEMENTATION NETWARK (GAIN)	Northern Ireland	No relevant content found	Regardless of age or living situation, the fundamental principles for maintaining optimal oral health remain consistent: practicing effective daily oral hygiene, minimising the intake of sugary foods and beverages, and attending regular dental check‐ups. It is also important to align dietary plans with the resident's oral health condition, avoiding excessive or frequent consumption of sugary foods and drinks when necessary. These recommendations are provided for nursing and care staff, residents, families and carers.	Managers and Care facilities are encouraged to follow Implement Regional Nutritional Guidelines for this age group. e.g., Ensure that regional nutritional guidelines for older people e.g., Promoting good Nutrition. A Strategy for good Nutritional Care for Adults in all Care Settings in Northern Ireland and Eat Well for Older People—Practical & Nutritional Guidelines for Food in Residential and Nursing homes and Community Meals (The Caroline Walker Trust).	A diminished oral function resulting in an inability to eat meat, fresh fruit and vegetables can lead to a reduced nutritional status, weight loss and poor recovery from illness and the associated concerns of malnutrition. A growing body of evidence now shows that poor oral health can have a significant impact on overall health.
OH3	National oral health improvement strategy for priority groups: frail older people, people with special care needs and those who are homeless	2012	Strategy	the Scottish Government	Scotland	No relevant content found	For caries prevention, care establishment managers should provide a variety of healthy food options that enable clients to reduce both the amount and frequency of sugary food consumption. Sugary foods should be limited to mealtimes, while also considering the specific nutritional needs of vulnerable individuals. To prevent dehydration, clients should be encouraged to choose water over beverages that contain sugar.	National Care Standards: Care Homes for Older People include 63 measures which are relevant to oral health. There is emphasis on a balanced nutritious diet within the standards but no specific reference to reducing sugar frequency. The standards require staff to regularly review anything that affects clients' ability to eat or drink, such as dental health, and to arrange for advice. There is also the need to maintain registration with a general dental practitioner and a recommendation that staff. The standards require that staff provide information about preventive healthcare, including screening.	Distinctions should be made between the nutritionally well and those who are nutritionally vulnerable and advice modified to take account of the patient's overall requirements.
OH 4	Oral health for adults in care homes. Information pack to support training.	2000	Toolkit	Public Health England, NHS Health Education England	England	Oral hygiene is important for good oral health but so is being aware of the number of times the resident has drinks and foods containing sugar. Older people could have a high sugar intake due to their medications or the diet they need. In these cases, good oral hygiene is even more important.	The more often in the day you eat food or drink containing sugar, the more likely there will be caries. By keeping foods and drinks containing sugar to mealtimes this allows the teeth time to recover and remineralise. Avoid sugary products just before bedtime as the saliva flow in the mouth slows down when you sleep, and can increase the caries risk. Low sugar, tooth‐friendly were recommended. However, reducing sugar intake This may not be appropriate for care home residents as a high proportion of residents are likely to be nutritionally vulnerable and at increased risk of dehydration.	No relevant content found	Evidence shows that poor oral health in older people can lead to problems chewing and swallowing which limit food choices and can lead to impaired nutritional status. Additionally, diet plays an important role in caries prevention.
OH 5	Caring for Smiles Guide for Care Homes. Better oral care for dependent older people	2013	Guideline	NHS Health Scotland, SCOTTISH GOVERNMENT, Care inspectare	Scotland	Many of the drinks that care home residents need or prefer contain high levels of sugar. Care home residents are at risk from dehydration and under‐nutrition and may need (or prefer) a higher intake of food and drinks with sugar. Older people in care homes are at increased risk of dehydration. A high proportion of residents are also likely to be nutritionally vulnerable. It is therefore important that oral health advice is given with a proper understanding of the dietary needs and risks of this group.	Caries are prevented mainly by reducing the number of times that acid attacks occur, for example by trying to keep sugar‐containing foods and drinks to mealtimes when possible. If older individuals require or prefer a higher intake of sugary foods or drinks, oral care should be improved accordingly. Enhanced mouth care includes brushing the teeth more often, or using high fluoride toothpaste or mouthwashes on the advice of a dentist. Collaborating with other professions, such as dietitians and speech and language therapists are recommended.	Nutritional risk assessment and Food in Hospitals in Scotland (Scottish Government, 2008) were introduced	Detailed mechanism of dental caries with sugar. Poor oral health impacts overall health, nutrition, QoL, communication and physical appearance. With the growing population of vulnerable older adults in care homes, insufficient oral care can negatively affect their nutrition and hydration status. Badly fitting dentures make eating difficult which increases the risk of under‐nutrition.
OH 6	Smile better Greater Manchester Mouth Care Toolkit	2016	Toolkit	Greater Manchester Combined Authority (GMCA), NHS in Greater Manchester	England	Many people will be on a high calorie diet to avoid or manage malnutrition. If high sugar drinks (e.g., nutritional supplements, fruit juices and squash, malted drinks) are drunk as quickly as possible and through a straw this will minimise the impact on oral health.	Mentioned about excessive and/or frequent sugar intake as a cause of caries. Cries can be reversed in the early stages by a good diet and fluoride. Keeping sugary foods and drinks to mealtimes, fluoride varnish and high fluoride toothpaste can help to prevent caries, good mouth care with a high fluoride toothpaste. Sugary food should be kept to mealtime and avoided at bedtime. Drinks and snacks between meals should be sugar‐free. If older people need medication in a liquid form then sugar‐free alternatives should be requested.	No relevant content found	People with good oral health can stay independent for longer and recover from episodes of frailty more quickly if they are able to eat and drink properly and take part in life.
OH 7	Blackburn with Darwen Oral Health Improvement Partnership Strategy 2021–2026	2022	Strategy	Blackburn with Darwen Borough Council	England	No relevant content found	No relevant content found	This guidance states that ‘Every resident's/client's hydration and nutrition should be reviewed regularly and included in their care plan. The care home should have a nutritional screening policy in place with one staff member taking responsibility for this policy within the home,’ emphasising the need for structured policies for nutritional screening in care homes. Additionally, it highlights the importance of training and professional development for staff employed by social care providers, which is critical in promoting good nutrition for older people. However, it does not elaborate on specific nutritional recommendations.	Good oral health supports proper eating and drinking, promoting independence and faster recovery from frailty. Conversely, poor oral health can cause pain, discomfort, and difficulties in eating and swallowing, reducing QoL. Additionally, certain prescription medications, especially when combined, may lead to oral issues such as thrush and dry mouth, impairing swallowing and potentially resulting in malnutrition.

Secondly, eligibility assessment was performed through title‐abstract screening by the two reviewers (SM and AS) while applying the inclusion and exclusion criteria. As abstracts are often not available for guidelines and policy documents, the reviewers also screened the literature by using executive summaries, or tables of contents, whichever was available in the literature. Finally, the full texts of the remaining included documents were screened by the two reviewers (SM and AS).

## 3 Results

The initial search identified 557 documents in total and after duplicates were removed, 476 remained. Screening of these documents by titles and abstracts further eliminated irrelevant documents and after this step, a full‐text search was performed for the 53 documents. A further 41 documents were excluded. Finally, of the 12 documents included in this scoping review: Four were classified as guidelines, seven as policy documents and one as a combined guideline–checklist. Policy documents comprised a range of formats, including guidance documents, checklists, strategies, toolkits and guides. All documents addressed either oral health considerations within nutrition‐focused content or nutrition‐related information within oral health materials. There were five nutritional documents that included oral health information [39, 40, 41, 42, 43] and seven oral health documents with nutritional/dietary information [44, 45, 46, 47, 48, 49, 50], as summarised in Tables 2 and 3 and shown in Figure 1. All documents were published in the UK between 2000 and 2024.

The following four headings categorise the outputs from the scoping review on the major common themes which emerged from the included documents: (1) The High‐sugar Diets in Care Homes, (2) Practical, Preventive Oral Health Guidance in Care Homes, (3) Cross‐Referencing of Nutritional and Oral Health Documents and Related Resources and (4) The Link Between Nutrition and Oral Health.

### 3.1 The High‐Sugar Diets in Care Homes

Nutritional documents NT1, NT2 and NT4 [39, 40, 42] and oral health documents OH4–OH6 [47, 48, 49] address the implications of sugar intake in care home residents, highlighting both its necessity—for example, in residents with reduced appetite, dementia‐related taste changes or those requiring fortified diets—and its associated risks. While both sets of documents acknowledge that sugar intake plays a significant role in dietary habits, with some NT2, NT4 and OH4 documents [40, 42, 48] noting that sources such as syrup‐based medications and oral nutritional supplements (ONSs) contribute to higher intake, their primary focus differs. Nutritional documents NT1, NT2 and NT4 [39, 40, 42] tend to emphasise the role of sugar in maintaining adequate energy intake for nutritionally vulnerable residents, whereas oral health documents OH4–OH6 [47, 48, 49] concentrate on strategies to limit sugar consumption and mitigate its impact on oral health.

#### 3.1.1 Oral Nutritional Supplements and Sugar Exposure

ONSs are mentioned in three nutritional documents NT1, NT2 and NT4 [39, 40, 42] and in oral health documents, OH2, OH4–OH6 [44, 47, 48, 49]. These documents note the high sugar content of ONSs (e.g., Fortisip and Ensure, as specifically mentioned in OH4 [47]), as well as that of some liquid medications.

Nutritional guidance NT1 [39] states that, for residents with poor appetite or dementia, food and drinks containing more sugar, including nutrition supplements, may be offered. NT4 [42] links high sugar intake from ONSs to root caries risk. Oral health guidance OH1 [44] notes that oral food supplements prescribed to maintain nutritional status can pose challenges to oral health in dentate residents in care homes. Additional oral health documents OH4–OH6 [47, 48, 49] provide guidance on enhanced oral care for residents consuming ONSs or other high‐sugar drinks, including advice on limiting intake to mealtimes, using straws when safe, and employing high fluoride products where appropriate.

In addition, excessive sugar consumption is identified as a potential risk factor for oral health deterioration. Documents NT2, NT4, OH5 and OH6 [40, 42, 48, 49] highlight the association between high sugar intake and dental issues such as caries, periodontal disease, and tooth loss.

Both nutritional and oral health documents also acknowledge that polypharmacy and conditions such as Candida infections may contribute to dry mouth, which some residents attempt to relieve by consuming sugary foods or drinks (e.g., sweets, tea or coffee with sugar). This is supported by NT4 [42], which also references oral health guideline OH5 [48], noting that care home residents at risk of dehydration and undernutrition may need (or prefer) a higher intake of food and drinks with sugar, emphasising the importance of enjoyment of food and the need for tailored nutrition advice.

#### 3.1.2 Differences in the Emphasis of Nutritional and Oral Health Documents

While both sets of documents discuss sugar intake, their emphasis differs. Nutritional documents NT1 and NT2 [39, 40] primarily focus on the role of sugar in supporting dietary needs, particularly among residents with reduced appetite or specific medical conditions. In addition, NT2 and NT4 [40, 42] highlight the potential risks of excessive sugar intake, such as higher risk of dental caries, including root caries, periodontal disease and tooth loss. In contrast, oral health documents OH4 and OH5 [47, 48] focus more on the preventive aspects of managing sugar consumption to mitigate its impact on oral health.

#### 3.1.3 Oral Hygiene Measures in Response to High Sugar Intake

An oral health toolkit OH4 [47] emphasises the importance of monitoring the frequency of sugar intake and highlights oral hygiene as a crucial measure to counteract the effects of high sugar exposure. Another toolkit OH6 [49] specifically recommends enhanced oral hygiene practices, including regular oral health assessments and staff training, as key strategies to mitigate the negative effects of high sugar intake on oral health. In contrast, nutritional documents NT2 and NT4 [40, 42], while recognising the oral health risks, do not propose specific preventive measures but rather outline the potential consequences of excessive sugar consumption, such as increased risk of dental caries, including root caries, periodontal diseases and tooth loss.

### 3.2 Practical, Preventive Oral Health Guidance in Care Homes

All nutritional documents NT1–NT5 [39, 40, 41, 42, 43] and oral health documents OH1–OH6 [44, 45, 46, 47, 48, 49] included in this review, except for the oral health strategy published in England in 2022 (OH7) [50], provide recommendations on preventive oral health strategies in care homes. These documents particularly focus on reducing sugar intake and improving oral hygiene practices among residents. While nutritional documents NT1–NT4 [39, 40, 41, 42, 43] address oral health concerns primarily in relation to dietary intake, oral health documents OH1–OH6 [44, 45, 46, 47, 48, 49] provide more specific guidance on caries prevention strategies, oral hygiene routines, and professional collaboration. The 2022 strategy (OH7) [50] is notable as it addresses care home residents' oral health primarily through policy adoption (in line with NICE NG48) rather than by providing its own detailed preventive strategies, highlighting a gap compared with other reviewed documents.

#### 3.2.1 Sugar Intake and Caries Prevention Strategies

Several nutritional documents NT1–NT3 [39, 40, 41] and oral health documents OH2–OH6 [45, 46, 47, 48, 49] emphasise that the frequency of sugar consumption is a key factor in caries development, recommending that sugary foods and drinks be restricted to mealtimes to minimise the number of acid attacks on teeth [39, 40, 45, 47, 48, 49]. Additionally, nutritional documents NT1 and NT2 [39, 40] and oral health document OH6 [49] highlight the association between ONSs and higher caries risk in the context of consuming various forms of fruit (e.g., fresh fruit, fruit juice and dried fruit) [39, 40, 49]. Oral health strategy OH3 [46] further recommends choosing water over sugar‐containing beverages to prevent dehydration while minimising caries risk.

Some nutritional documents NT1 and NT2 [39, 40] note that reducing sugar intake may not always be feasible in care home residents due to their malnutrition status and the need for energy‐dense diets. Oral health document OH4 [47] similarly mentions that dietary planning should consider both oral health and nutritional needs.

#### 3.2.2 Oral Hygiene Recommendations

In response to the potential risks posed by high sugar intake, nutritional documents NT4 and NT5 [42, 43] and oral health documents OH1, OH5 and OH6 [44, 48, 49] recommend enhanced oral hygiene practices. Documents NT1, NT2, NT4, OH1 and OH4–OH6 [39, 40, 42, 44, 47, 48, 49] suggest that residents who require ONSs, sugary foods and drinks, or sugar‐containing medications should receive extra oral care to mitigate potential risks of caries and/or gum disease. Specific recommendations include brushing teeth more frequently, using high‐fluoride toothpaste or mouthwash as advised by a dentist, and applying fluoride varnish as a protective measure [48, 49]. Additionally, care home staff are encouraged to support residents in maintaining daily oral hygiene routines as part of their personalised care plans [43].

Nutritional guideline NT3 [41] recommends the involvement of dentists in the establishment of nutrition and hydration care plans, recognising their role in providing guidance on food selection and meal timing. One oral health guideline OH5 [48] also reference interdisciplinary collaboration, including coordination with dietitians, speech and language therapists, and care home managers. Additionally, staff training in oral health management is mentioned in one guidance NT4 [42] as a component of overall care provision in care homes.

### 3.3 Cross‐Referencing of Nutritional and Oral Health Documents and Related Resources

#### 3.3.1 Nutritional Documents Referencing Oral Health Resources

Several nutritional documents included in this review reference established oral health programs and guidelines as resources for improving oral health care in care home settings. Nutritional guidance NT1 [39] introduces the Gwên am Byth programme [51], a national initiative in Wales aimed at ensuring consistent, high‐quality oral hygiene and mouth care for care home residents. It also suggests that nutrition care plans should be supported by an oral health risk assessment, such as the All Wales Oral Health Risk Assessment included in the Gwên am Byth programme.

Another nutritional guidance NT5 [43] provides an extensive list of oral health guidelines and resources, including national and international materials such as the National Institute for Health and Care Excellence (NICE)'s ‘Oral Health for Adults in Care Homes’ [52] and dementia‐specific mouth care guidance from Dementia UK. Separately, NT4 [42] references the *Caring for Smiles Guide for Care Homes*, a Scottish national oral health guideline that provides guidance on oral hygiene, risk assessments, and oral care strategies for residents. NT4 [42] also highlights the importance of integrating oral health practices into broader care home policies and emphasises the need for staff training on oral health management. Furthermore, NT5 [43] refers to NICE NG48: *Oral Health for Adults in Care Homes* [52] as a relevant source of an oral health guideline but does not elaborate on the specific content of this guideline.

#### 3.3.2 Oral Health Documents Referencing Nutritional Policies

Several oral health documents OH1–OH3, OH5, OH7 [44, 45, 46, 48, 50] emphasise the importance of incorporating nutrition‐related considerations into care home management. Oral health guideline OH1 [44], issued by the Nutrition Task Force, underscores the need to provide older adults with clear, practical, and achievable advice on selecting a balanced diet.

Another oral health document OH2 [45] recommends aligning care home practices with regional nutritional guidelines, such as *Promoting Good Nutrition—a Strategy for Good Nutritional Care for Adults in All Care Settings in Northern Ireland* [53] and *Eat Well for Older People—Practical & Nutritional Guidelines for Food in Residential* and *Nursing Homes and Community Meals* [54].

Oral health strategy OH3 [46] discusses *the National Care Standards: Care Homes for Older People* [55], which include 63 measures related to oral health. These standards require regular review of factors affecting residents' ability to eat and drink, including oral health, and recommend that staff provide preventive healthcare information. However, the *National Care Standards* [55] do not specifically mention reducing sugar intake as a strategy for oral health improvement.

Another oral health guideline OH5 [48] refers to nutritional risk assessment policies and the Food in Hospitals guidance issued by the Scottish Government. Another oral health strategy OH7 [50] also highlights the importance of hydration and nutrition assessments for all residents, recommending that care homes implement structured policies for nutritional screening and that a designated staff member be responsible for overseeing this process. OH7 [50] strategy also underscores the importance of staff training and professional development to ensure the promotion of good nutrition among older people. However, it does not provide specific nutritional recommendations.

### 3.4 The Link Between Nutrition and Oral Health

#### 3.4.1 Impact of Poor Oral Health on Nutrition

Both nutritional documents NT1, NT4 and NT5 [39, 42, 43] and oral health documents OH1, OH2, OH4, OH5 and OH7 [44, 45, 47, 48, 50] included in this review highlight the strong interconnection between oral health and nutrition among older adults in care homes. These documents emphasise that poor oral health can lead to difficulties in chewing, swallowing, and tasting food, which in turn may result in reduced dietary intake, malnutrition and overall health deterioration [39, 42, 43, 44, 45, 47, 48, 50]. Additionally, multiple documents NT1, NT5, OH2 and OH5 [39, 43, 45, 48] state that ill‐fitting dentures or sore gums can contribute to dietary restrictions, particularly affecting the consumption of nutrient‐dense foods such as fruits, vegetables, and meat.

#### 3.4.2 Role of Oral Health in Supporting Independence and Functional Ability

Several documents NT4, OH6 and OH7 [42, 49, 50] highlight that good oral health supports proper eating and drinking, which is essential for maintaining independence, promoting social engagement and enhancing functional ability in older adults. Some documents NT4 and OH7 [42, 50] further indicate that older adults with dementia may face additional challenges related to eating and drinking due to oral health issues and swallowing difficulties, increasing their risk of malnutrition.

#### 3.4.3 Importance of Hydration in Oral and Nutritional Health

Both nutritional NT5 [43] and oral health OH7 [50] documents acknowledge the role of hydration in maintaining oral and overall health. Inadequate hydration is linked to an increased risk of oral conditions such as dry mouth, which may further impair swallowing and dietary intake. The oral health strategy OH7 [50] specifies that certain prescription medications, particularly when combined, can contribute to oral health problems such as thrush and dry mouth, potentially leading to nutritional deficiencies.

#### 3.4.4 Dietary Recommendations and Oral Health

Regarding dietary recommendations, one nutritional document NT3 [41] suggests that all fluids contribute to hydration but recommends water, tea, coffee (without added sugar) and milk as preferable options for maintaining oral health. Additionally, oral health documents OH4 and OH5 [47, 48] recognise that diet plays an important role in caries prevention, though they emphasise the need to balance nutritional considerations with oral health risks.

#### 3.4.5 Screening for Oral Health‐Related Nutritional Risks

Furthermore, nutritional guidance NT1 [39] specifically recommends identifying weight loss in residents with ill‐fitting dentures during initial care assessments, as this may indicate previous nutritional deficiencies. Another document NT2 [40] notes that with an increasing proportion of older adults retaining their natural teeth, maintaining oral health contributes positively to general health, nutritional status and QoL.

## 4 Discussion

### 4.1 Overview and Scope of the Review

An important finding of this review is that although 12 relevant documents were identified, most primarily addressed either nutrition or oral health, with limited integration of both domains. Furthermore, the publication years of the included documents revealed notable variation in recency. Half of the documents (6 out of 12) were published more than a decade ago, particularly five of the seven oral health documents [40, 44, 45, 46, 47, 48]. This raises concerns about whether these documents adequately reflect current evidence and best practices, especially given the evolving understanding of the relationship between nutrition and oral health in older adults. In contrast, nutritional documents have been updated more frequently [39, 41, 42, 43], with only one published before 2014 [40]. This discrepancy suggests that while nutrition‐related policies are being revised periodically, oral health policies may lag behind, potentially limiting opportunities to develop integrated approaches to care. Given the increasing recognition of the interplay between oral health and nutrition, there is an urgent need for more comprehensive and up‐to‐date guidelines and policy documents that systematically incorporate both domains for older adults in care settings.

### 4.2 High‐Sugar Diets in Care Homes and Oral Health Implications

Both nutritional and oral health guidelines and policy documents acknowledge the significant role sugar intake plays in the dietary habits of older care home residents. While the necessity of sugar‐containing foods and drinks is recognised for individuals with reduced appetite or specific medical conditions, including those requiring fortified diets [39, 40], the potential long‐term consequences on oral health are often underemphasised in nutritional documents. In contrast, oral health documents primarily focus on the preventive aspects of sugar intake management to mitigate oral health deterioration [47, 48], highlighting the need for a more balanced approach that considers both nutritional needs and oral health risks.

A key concern is the lack of clear, practical guidance on how to mitigate the oral health risks associated with high‐sugar intake, ONSs and syrup‐based medications. While oral health documents acknowledge these risks, they often lack specific recommendations on how care homes should balance sugar intake with oral health protection [44, 47, 48, 49]. Similarly, nutritional documents recognise sugar's impact on oral health but rarely incorporate structured oral hygiene strategies to minimise its adverse effects [39, 40, 41, 42]. This disconnect suggests a gap in interdisciplinary coordination, which could be addressed by integrating dietary and oral health management strategies into a unified guideline and/or policy document.

Furthermore, the emphasis on sugar as an energy source for nutritionally vulnerable older adults may inadvertently contribute to increased oral health problems, particularly in those with existing dental issues or polypharmacy‐induced dry mouth [42, 47, 48, 49]. Future guidelines and/or policy documents should aim to reconcile these competing priorities by offering evidence‐based recommendations that support both adequate nutrition and oral disease prevention.

### 4.3 Practical, Preventive Oral Health Guidance in Care Homes

Most of the reviewed documents (except for the oral health strategy published in England in 2022 [50]) provide recommendations on preventive oral health strategies in care homes [39, 40, 41, 42, 43, 44, 45, 46, 47, 48, 49]. These strategies focus on reducing sugar intake and improving oral hygiene. Oral health documents offer more specific recommendations, including limiting sugary foods and drinks to mealtimes, enhanced oral hygiene measures and professional collaboration to mitigate caries risks [45, 46, 47, 48, 49].

While these recommendations provide a strong foundation for oral health management, their practical implementation presents several challenges. First, there is a lack of consistency across documents regarding sugar restriction. Some documents strongly advocate for limiting sugar intake to mealtimes [45, 46], whereas others acknowledge that nutritionally vulnerable older adults may require additional sugar intake due to reduced appetite or medical conditions [39, 42]. This discrepancy raises questions about how best to balance oral health concerns with the dietary needs of care home residents.

Furthermore, while enhanced oral hygiene is recommended, its feasibility in care home settings remains uncertain. Staff shortages, limited training in oral care, and the resistance of some residents, particularly those with dementia, may hinder the consistent application of oral hygiene measures [42, 48]. Without adequate support, these documents may fail to translate into meaningful improvements in residents' oral health.

To address these challenges, several documents emphasise the importance of interdisciplinary collaboration in care homes [41, 48]. Recommendations include involving dentists in care planning, coordinating with dietitians to balance dietary needs with oral health considerations and integrating speech and language therapists to support residents with swallowing difficulties [41, 48]. However, despite these recommendations, many documents lack clear guidance on how different healthcare professionals should collaborate in practice. In many care homes, staff often have limited access to dental professionals, making it difficult to implement comprehensive oral health strategies.

Additionally, the emphasis on sugar reduction must be weighed against QoL considerations. For many older adults, especially those with cognitive impairment, food enjoyment is an essential aspect of well‐being. Strict dietary restrictions could potentially reduce overall food intake and diminish their QoL [56]. Therefore, a more balanced approach—one that considers both oral health and nutritional needs—is necessary to develop practical and sustainable oral health strategies in care homes.

### 4.4 The Bidirectional Relationship Between Nutrition and Oral Health

The relationship between nutrition and oral health is widely recognised as bidirectional, with each influencing the other in complex ways. Both nutritional and oral health documents emphasise that poor oral health can contribute to malnutrition, and vice versa [39, 42, 43, 44, 45, 47, 48, 50]. However, despite this recognition, existing documents often address these domains separately rather than as interconnected aspects of health, potentially limiting the effectiveness of interventions.

One major concern is the impact of oral health deterioration on nutritional intake. Chewing and swallowing difficulties, often due to ill‐fitting dentures, periodontal disease or tooth loss, can restrict dietary choices and lead to nutritional deficiencies [39, 43, 45, 48]. While some documents highlight the need to accommodate these challenges by modifying food texture and offering nutrient‐dense alternatives [42, 50], they provide limited guidance on integrating oral health interventions with dietary recommendations. In practice, care home staff may lack the necessary training to assess how oral health issues impact nutritional intake, leading to unintended dietary restrictions that further exacerbate malnutrition risk [57, 58].

Conversely, nutritional deficiencies can negatively affect oral health, creating a cycle of decline. Inadequate dietary intake can impair immune function and reduce saliva production, increasing susceptibility to dental caries, periodontal disease and oral infections [42, 50]. Older adults with dementia are particularly vulnerable, as cognitive impairments can lead to irregular eating habits, reduced oral hygiene and increased reliance on sugar‐containing foods and beverages [42, 50]. Despite these risks, many documents fail to offer specific strategies for preventing nutritional deficiencies that could compromise oral health outcomes.

Moreover, the current approach to oral health management in care homes often focuses on hygiene rather than dietary interventions [42, 43, 50], potentially overlooking opportunities for more holistic care. While enhanced oral hygiene is important for preventing oral diseases, it does not address the underlying nutritional factors that contribute to poor oral health [59]. Integrating oral health assessments with routine nutritional screenings could allow for more proactive interventions, such as adjusting diet plans to improve both oral and systemic health outcomes [60].

Given these challenges, a more integrated approach to oral health and nutrition is necessary. Future guidelines and policy documents should emphasise the interdependence of these factors and promote interdisciplinary collaboration between dietitians, dentists and care staff. Additionally, more practical recommendations on adapting dietary plans for residents with oral health issues are needed to ensure that interventions are both nutritionally adequate and supportive of oral function.

By shifting from a siloed approach to a more integrated model, care homes could better support the health and well‐being of older adults, preventing the cascading effects of poor oral health and malnutrition. Addressing this bidirectional relationship holistically could improve not only individual outcomes but also overall care quality in residential settings.

### 4.5 Limited Inclusion of Oral Health in Nutritional Guidelines and Policy Documents

Despite the well‐documented interplay between oral health and dietary intake [19, 61], nutritional guidelines and policy documents do not always provide detailed guidance on oral health considerations. The majority primarily focus on meeting dietary needs but do not incorporate specific measures to prevent oral diseases [39, 40, 42]. While some documents acknowledge the risks associated with high sugar intake [40, 42], they generally fail to integrate practical strategies that balance both nutritional and oral health needs.

This gap suggests that oral health remains undervalued as a component of nutritional well‐being in care homes. Poor oral health can limit food choices, reduce appetite and cause discomfort during eating [61, 62], yet the lack of specific oral health recommendations in nutritional documents may hinder comprehensive care. Furthermore, many older residents rely on modified diets or ONSs [63], and while these documents acknowledge their potential impact on oral health and propose strategies to mitigate associated risks, such strategies are generally limited in scope [39, 40, 42].

The fragmented approach between oral health and nutrition also leads to inconsistencies in care. While oral health documents advocate sugar reduction and oral hygiene [45, 46, 47], these considerations are not consistently reflected in nutritional policies, potentially undermining oral health efforts. To enhance care home policies, future guidelines and policy documents should integrate oral health as a key consideration, providing specific recommendations on food selection, sugar intake management and interdisciplinary collaboration. A more holistic approach could ensure that dietary strategies support both adequate nutrition and oral health, ultimately improving residents' well‐being and QoL.

### 4.6 Limited Inclusion of Nutrition in Oral Health Guidelines and Policy Documents

Oral health guidelines and policy documents primarily emphasise caries prevention through sugar restriction and oral hygiene [47, 48]. However, they often overlook the role of balanced nutrition in maintaining oral and systemic health [61], missing an opportunity for a more comprehensive approach in care homes.

For example, NG48, NICE's oral health guideline for care homes, focuses on oral hygiene but provides little practical guidance on how dietary strategies could be incorporated into oral health management [52]. However, this guideline was not included in our final selection following the screening process and was therefore not analysed in the present study. The absence of dietary guidance may lead to frequent and excessive sugar intake, increasing caries risk.

Moreover, while many oral health documents stress sugar restriction [45, 46, 47, 48, 49], they do not address how to implement these measures without compromising nutritional adequacy for older adults. In care homes, where residents often have reduced appetite, chewing difficulties or increased reliance on ONSs, a strict focus on sugar avoidance without alternative dietary strategies may inadvertently contribute to nutritional deficiencies [64, 65]. This underscores the need for guidelines and policy documents that balance caries prevention with broader nutritional needs.

Future oral health guidelines and policy documents should expand beyond hygiene‐based recommendations to incorporate evidence‐based nutritional strategies. Encouraging collaboration between oral health professionals, dietitians and care home staff could enhance both dietary intake and oral health outcomes. Integrating these aspects in updated guidelines and policy documents would promote a more effective and sustainable model of care for older adults in institutional settings.

### 4.7 Strengths, Limitations, and Future Research

This scoping review systematically identified and analysed existing UK‐based guidelines and policy documents addressing both oral health and nutrition in care homes. One strength of this review is its comprehensive search strategy, which captured a broad range of guidelines and policy documents beyond traditional academic literature. However, a limitation of this study is the exclusion of international guidelines and policy documents, which may offer further insights into integrated approaches to nutrition and oral health.

Future research should focus on developing and validating a comprehensive oral health‐nutritional guideline and/or policy document tailored for care home settings. Such a guideline and/or policy documents should incorporate best practices from both disciplines, address the specific dietary needs of older adults while minimising oral health risks, and provide clear recommendations for interdisciplinary collaboration. Establishing evidence‐based policies that integrate oral health and nutrition will be essential in improving the overall health and well‐being of older adults residing in care homes.

## 5 Conclusion

This scoping review highlights the lack of integration between oral health and nutritional guidelines and policy documents in UK care homes. While nutritional documents focus on dietary adequacy with minimal consideration of oral disease prevention, oral health documents emphasise sugar restriction and hygiene but overlook broader nutritional needs. This fragmented approach may compromise care quality, particularly for older adults relying on modified diets or ONSs.

To enhance care effectiveness, future guidelines and/or policy documents should adopt a holistic framework that aligns dietary strategies with oral health preservation. Strengthening interdisciplinary collaboration and incorporating practical recommendations for balancing sugar intake with oral health needs will be essential in improving the overall well‐being of care home residents.

## Author Contributions

Sachi Makino conceived the study, designed the review protocol, performed the primary screening, data extraction, and analysis, and drafted the manuscript. Aziza Sallam served as the second reviewer, contributing to screening and validating the extracted data. Gerry McKenna and Jayne Woodside supervised the process and critically reviewed the manuscript. All authors approved the final version of the manuscript.

## Funding

The authors have nothing to report.

## Ethics Statement

The authors have nothing to report.

## Consent

The authors have nothing to report.

## Conflicts of Interest

The authors declare no conflicts of interest.

## Data Availability

Data sharing not applicable to this article as no datasets were generated or analysed during the current study.
